# Evaluating the Impact of a Biomimetic Mechanical Environment on Cancer Invasion and Matrix Remodeling

**DOI:** 10.1002/adhm.202201749

**Published:** 2022-11-20

**Authors:** Auxtine Micalet, Judith Pape, Deniz Bakkalci, Yousef Javanmardi, Chloe Hall, Umber Cheema, Emad Moeendarbary

**Affiliations:** ^1^ Department of Mechanical Engineering University College London Gower Street London WC1E 6BT UK; ^2^ UCL Centre for 3D Models of Health and Disease Department of Targeted Intervention Division of Surgery and Interventional Science University College London Charles Bell House 43–45 Foley Street London W1W 7TS UK

**Keywords:** 3D tumor models, atomic force microscopy, cancer invasion, MMP, mechanobiology, stiffness, tumor microenvironments

## Abstract

The stiffness of tumors and their host tissues is much higher than most hydrogels, which are conventionally used to study in vitro cancer progression. The tumoroid assay is an engineered 3D in vitro tumor model that allows investigation of cancer cell invasion in an environment that is biomimetic in terms of extracellular matrix (ECM) composition and stiffness. Using this model, the change in matrix stiffness by epithelial colorectal cancer cells is systematically characterized by atomic force microscopy indentation tests. Less invasive epithelial cancer cells stiffen the tumor microenvironment while highly aggressive epithelial cancer cells show significant softening of the tumor microenvironment. Changes in stiffness are attributed to both cell‐generated active forces as well as ECM degradation and remodeling. The degradation is in part attributed to the enzymatic activity of matrix metalloproteinases (MMPs) as demonstrated by the significant expression of MMP‐2 and MMP‐9 at both gene and protein levels. Targeting MMP activity through broad‐spectrum drug inhibition (BB‐94) reverses the changes in stiffness and also decreases cancer cell invasion. These results promote the idea of using mechano‐based cancer therapies such as MMP inhibition.

## Graphical abstract

Using a biomimetic 3D cancer model, the role of cancer cells on tissue stiffness is investigated. It is observed through atomic force microscopy stiffness measurements that less invasive colorectal cancer cells stiffen the matrix while highly invasive colorectal cancer cells soften the matrix. Targeting MMP through drug inhibition downregulates matrix remodelling. Inhibiting matrix degradation decreases cancer cell invasion, implicating that mechano‐based therapy can be an efficient treatment.

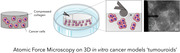

## 1 Introduction

Cells sense their physical microenvironment through mechanoreceptors, mainly through the transmembrane protein family integrins.^[^ ^1^, ^2^ ^]^ Biomechanical cues trigger stiffness‐dependent cell behavior. Mesenchymal stem cells differentiate down different lineages depending on substrate stiffness.^[^ ^3^ ^]^ Normal fibroblasts have been shown to undergo more apoptosis and less proliferation on soft substrates compared to stiffer substrates.^[^ ^4^ ^]^ These examples highlight the importance of integrating a biomimetic mechanical microenvironment into any in vitro model.

Tissue stiffness also plays a major role in cancer progression.^[^ ^5^ ^–^ ^12^ ^]^ As the tumor grows, the cancer cells induce matrix remodeling, which in turn promotes cell invasiveness. TGF‐*β* gets activated in new cancer cells which then leads to matrix stiffening.^[^ ^13^, ^14^ ^]^ The increased local stiffness affects intracellular signaling, by modifying integrins.^[^ ^13^ ^]^ This, in turn, triggers an upregulation of mesenchymal markers and downregulation of epithelial markers, such as E‐cadherin, leading to an epithelial‐to‐mesenchymal transition (EMT).^[^ ^12^, ^15^, ^16^ ^]^ As the cancer progresses, unique remodeling processes occur, attributed to the actions of proteins such as lysyl oxidase (LOX),^[^ ^17^ ^–^ ^21^ ^]^ metallopeptidase inhibitors (TIMPs),^[^ ^22^ ^]^ and matrix metalloproteinases (MMPs).^[^ ^23^, ^24^ ^]^ Perturbing the distinctive remodeling pattern, in the hopes of limiting cancer progression is the goal of the emerging field of mechano‐based therapies.^[^ ^25^ ^]^ MMPs are therapeutic targets with over 50 MMP inhibitors being investigated.^[^ ^26^ ^]^

**Figure 1:**
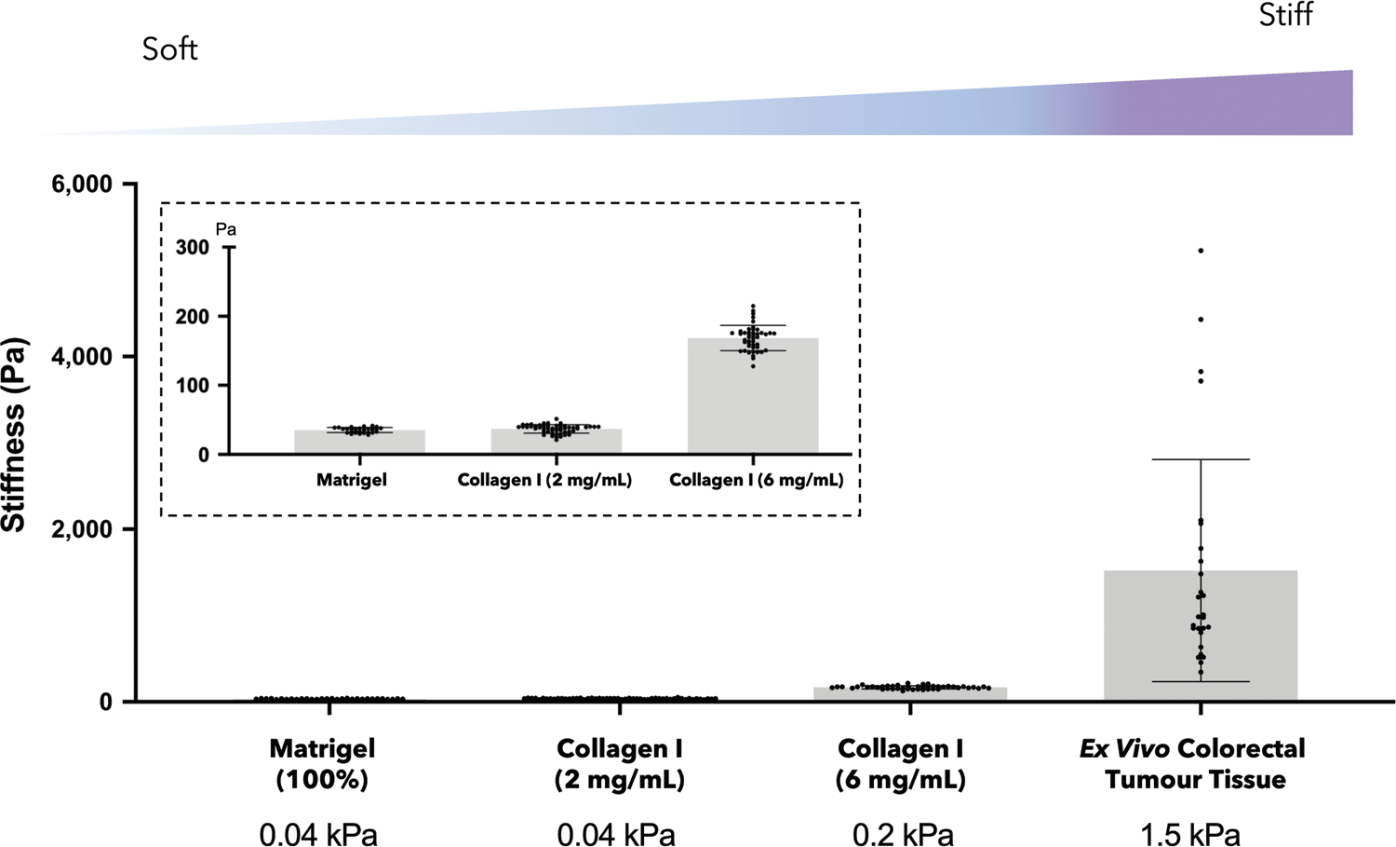
Stiffness of various 3D hydrogel models compared to ex vivo tumor tissue. Atomic force microscopy (AFM) measurements of the stiffness of Matrigel (100%), collagen I (2 and 6 mg mL^−1^) and ex vivo colorectal tumor tissue. The inset is a zoom of the hydrogels’ stiffness. 3D hydrogel models were measured as *n* = 3 samples, with >30 measurement points per sample. One colorectal tumor tissue was collected and AFM measurements were conducted on three sections with >10 measurement points per section.

To study cancer in vitro, appropriate replication of the biomechanical cues from the tumor microenvironment (TME), and particularly matrix stiffness, within 3D models is essential.^[^ ^5^ ^]^ Commonly used 3D models such as Matrigel or collagen hydrogels are orders of magnitude softer compared to the stiffness of most organs and tumor tissues. Indeed, we measured a seven‐fold difference between the mechanical properties of standard 3D models (Matrigel, collagen I at 2 and 6 mg mL^−1^) and ex vivo colorectal tumor tissue (**Figure** **1**). Various methods can be applied to better recapitulate tissue stiffness,^[^ ^27^, ^28^ ^]^ with the simplest strategy to varying collagen I gels material properties, such as concentration, polymerization temperature, and pH.^[^ ^29^ ^]^ Other techniques to stiffen collagen type I include chemical cross‐linking (glycation via glucose supplementation^[^ ^30^ ^]^ or LOX^[^ ^31^ ^]^). Using crowding agents, such as adding alginate to Matrigel,^[^ ^32^, ^33^ ^]^ alginate to collagen,^[^ ^34^ ^]^ or agarose to Matrigel,^[^ ^35^ ^]^ also allows tuning of matrix stiffness. Strain stiffening of collagen type I gels has been shown to increase their stiffness four‐fold.^[^ ^36^ ^]^ All of these scaffolds have been shown to directly impact invasion and metastasis in 3D cancer cultures. A recent study highlights that different facets of collagen architecture can influence cancer invasion in different ways.^[^ ^37^ ^]^ Collagen bundling was shown to enhance breast cancer invasion, compared to “systemic” collagen stiffening, which prevented collective cancer invasion.

**Figure 2:**
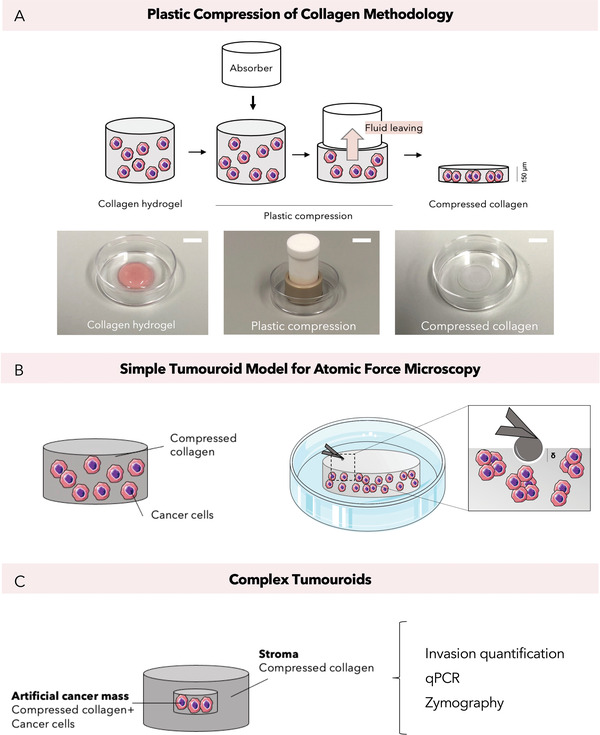
Major experimental methodologies. A) Plastic compression of collagen type I hydrogels using RAFT absorbers leading to dense compressed collagen gels. Scale bars = 10 mm B) AFM measurements were conducted on compressed collagen gels containing cancer cells (referred to as simple “tumoroids”). C) Invasion quantification, gene analysis and protein analysis were performed on complex “tumoroids.” These are compartmentalized tumoroid models that consisted of compressed collagen with cancer cells in a centeral “artificial cancer mass” surrounded by an acellular stroma.^[^ ^38^ ^–^ ^42^ ^]^

Within the scope of this study, we engineered a colorectal cancer model using compressed collagen (**Figure** **2**A). This 3D model, termed “tumoroid” is fabricated through plastic compression of a type I monomeric collagen hydrogel embedded with cancer cells.^[^ ^38^ ^–^ ^42^ ^]^ The compression removes excess liquid, resulting in a collagen dense matrix that more appropriately recapitulates the in vivo TME.^[^ ^38^ ^–^ ^42^ ^]^ The tumoroids can be cultured for extended duration (21 days in this study). Although other extracellular matrix (ECM) proteins can be added to the tumoroid model,^[^ ^40^, ^41^ ^]^ for this study only collagen I was used as it is the predominant ECM component in colon cancer tissues^[^ ^43^ ^]^ and its simple composition allows for a better understanding of the role of stiffness. Using this engineered 3D model, we investigated how cancer cells remodel a 3D matrix of physiologically relevant collagen density and stiffness. Furthermore, the tumoroid model was used to interrogate the possibility of controlling matrix remodeling to limit cancer invasion.

The mechanical properties were measured by atomic force microscopy (AFM) indentation of the tumoroid samples (Figure 2B). The indentation delta (in µm) and the force applied (in nN) can be correlated back to stiffness (Young's modulus, Pa). This differs from methods such as rheology, where only bulk stiffness can be measured. AFM can be used on biological samples^[^ ^44^ ^–^ ^46^ ^]^ and 3D in vitro models, although a specific methodology had to be developed to this aim.

## 2 Results and Discussion

### 2.1 The Tumoroid: A 3D Model of Biomimetic Stiffness

**Figure 3:**
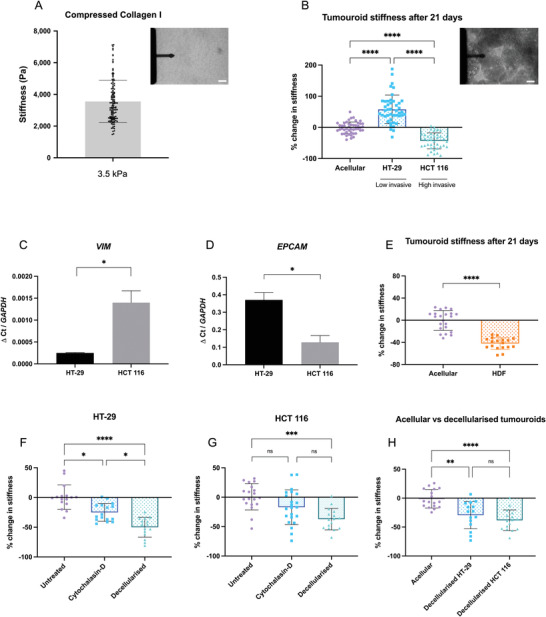
Effect of cancer cells on tumoroid stiffness over 21 days. A) Stiffness of acellular compressed collagen measured by AFM. Top right panel: AFM cantilever with glued 50 µm bead probing an acellular tumoroid. Scale bar = 100 µm B) Percentage change in the stiffness of HT‐29 and HCT 116 cellular tumoroids measured by AFM (*n* = 6, Kruskal–Wallis significance). Top right panel: AFM cantilever probing a 21 day cellular tumoroid. Scale bar = 100 µm C and D) EMT gene expression in the cancer cells after 21 days (EPCAM, epithelial marker; VIM, mesenchymal marker). Gene levels were normalized to GAPDH (*n* = 3, Unpaired *t*‐test significance). E) Percentage change in the stiffness of human dermal fibroblasts (HDF) cellular tumoroids measured by AFM (*n* = 3, Kruskal–Wallis significance) F and G) Stiffness of 21 days tumoroids, prior to and after 1 h cytochalasin‐D treatment or decellularization (*n* = 3, Kruskal–Wallis significance). H) Comparison of decellularized tumoroid stiffness with the stiffness of acellular condition (*n* = 3, Kruskal–Wallis significance). All *p*‐value significance is indicated as: 0.05 < *, 0.01 < **, 0.001 < ***, and 0.0001 < ****.

The stiffness of two commonly used 3D models (Matrigel and collagen I hydrogels), the tumoroid model, and ex vivo colorectal tumor tissue were measured and compared using AFM (Figure 1, **Figure** **3** **A**). All samples were measured using the same experimental setting with over 30 AFM measurements per sample type. The stiffness of Matrigel was 35 ± 4 Pa, collagen type I hydrogels (2 mg mL^−1^) were 37 ± 6 Pa, and collagen type I hydrogels (6 mg mL^−1^) were 168 ± 18 Pa. The stiffness of ex vivo colorectal tumor tissue was 1.5 ± 1.3 kPa, which is consistent with previous measurements conducted by Deptuła et al. using AFM reporting an average stiffness of 5.80 ± 3.8 kPa, over four colorectal cancer tissue samples.^[^ ^47^ ^]^ The stiffness of colorectal tumor tissue is therefore ≈40‐fold higher than the stiffness of the Matrigel and collagen (2 mg mL^−1^) hydrogels. The stiffness of the compressed collagen gel used for our tumoroid model was 3.5 ± 1.3 kPa, which is close to the stiffness of colorectal tumor tissue.

Due to variabilities in reagents, collagen batches, plastic compression times, pH, and gelation temperature/time and manual handling, we observed some variations in the stiffness of acellular control gels at different experimental days (see Figure S1, Supporting Information). To counter this variation, each experiment was set alongside an *n* = 3 of acellular gels, to which we then normalized the measured AFM data of cellular constructs. Therefore, all subsequent reported stiffness values were normalised to their individual acellular controls.

### 2.2 Effect of the Degree of Invasiveness of Cancer Cells on Their Remodeling Pattern

After 21 days the stiffness of tumoroids with either HT‐29 (less invasive colorectal cancer cells) or HCT 116 (high invasive colorectal cancer cells) was measured by AFM indentation (Figure 3B). It was observed that HT‐29 cells significantly stiffened the matrix (60% stiffening compared to acellular control, *p* < 0.0001), whilst more invasive cells (HCT 116) significantly softened the matrix (−43% softening compared to acellular control, *p* < 0.0001).

**Figure 4:**
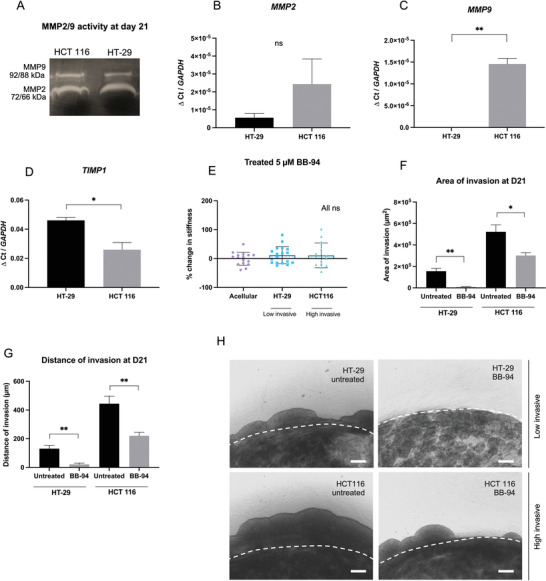
Broad‐spectrum MMP inhibition (BB‐94) limits matrix remodeling and cancer cell invasion. A) Zymography showing MMP‐2 and MMP‐9 presence and activity at day 21. B–D) Gene expression after 21 days of matrix remodelling markers (MMP‐2, MMP‐9, TIMP‐1). Gene levels normalized to GAPDH (*n* = 3, Unpaired *t*‐test significance). E) Percentage change in the stiffness of HT‐29 and HCT 116 tumoroids after 21 days of Batimastat treatment to inhibit MMPs (*n* = 3, Kruskal–Wallis significance). F,G) Distance and area of invasion of cancer cells from the artificial cancer mass into the stroma of a tumoroid. Untreated versus treated with 5 µm BB‐94 (*n* = 3, Unpaired *t*‐test significance). H) Imaging at day 21 of the cancer cells invading from the artificial cancer mass into the stroma. Scale bar = 200 µm. All *p*‐value significance is indicated as: 0.05 < *, 0.01 < **, 0.001 < ***, and 0.0001 < ****.

VIM mRNA expressed was significantly upregulated in the HCT 116 tumoroids (*p* = 0.0131, Figure 3C), while significant upregulation of EPCAM mRNA in HT‐29 tumoroids was measured (*p* = 0.0135, Figure 3E). This indicates a stronger mesenchymal and epithelial phenotype in HCT 116 and HT‐29 tumoroids, respectively. The differences in EMT status were later further explored through quantification of migratory and invasion behaviors. Greater distance and area of invasion in HCT 116 tumoroids compared to HT‐29 were observed (**Figure** **4**F–H). To demonstrate that softening the matrix is a mesenchymal characteristic, tumoroids with human dermal fibroblasts (HDF, which are mesenchymal cells) were set up. After 21 days, a similar pattern of significant softening was observed (−42.1% compared to acellular control, *p* < 0.0001, Figure 3E).

Considering the above data, we hypothesize that highly invasive cancer cells cleave collagen fibers and enlarge the pore size of the matrix to migrate and invade into the surrounding tissues.^[^ ^48^ ^]^ In contrast, less invasive cancer cells remodel and contract the collagen matrix to stiffen it, a reaction that creates a “shield,” and protects the cancer cells from external factors.^[^ ^48^ ^]^ These observations are consistent with the idea that ECM remodeling in tumor progression starts with ECM deposition (changing the abundance of ECM fibers and/or cross‐linking) and force‐mediated physical remodeling,^[^ ^49^ ^]^ and then followed by local proteolytic degradation, which may be required for opening‐up passages for cell migration.^[^ ^49^ ^]^

The stiffness patterns observed above using the 3D tumoroid model, correlate with observations made on biopsy samples.^[^ ^45^, ^50^ ^]^ Conklin et al. observed that early onset tumors had a desmoplastic reaction with collagen fibers that were aligned parallel to the tumor boundary. Later stage tumors, correlated with worse patient survival, had less dense and re‐orientated collagen fibers, thus promoting cell invasion.^[^ ^50^ ^]^ Plodinec et al. conducted AFM measurement on three types of breast tissue biopsy (normal tissue, benign tumor, and invasive tumor) and showed that late, invasive stage tissue has softer regions as a result of the cancer cells infiltrating the surrounding tissue.^[^ ^45^ ^]^ Taken together, our data from an in vitro model correlates with trends from ex vivo samples highlighting the importance of using a biomimetic matrix, when modeling diseases.^[^ ^51^, ^52^ ^]^

### 2.3 Dissecting the Contribution of Cell‐Generated Forces versus Matrix Remodeling

The structural mechanical properties of the ECM, mechanical properties of cells, and active cellular forces that contract the ECM, all contribute to the overall stiffness of cell embedded 3D matrices.^[^ ^6^, ^8^, ^53^ ^–^ ^55^ ^]^ Cells indeed actively generate tension within a 3D environment by pulling on and contracting the ECM fibers.^[^ ^56^, ^57^ ^]^ We therefore aimed to apportion the contribution of cell‐generated contraction and ECM remodeling (ECM reorganization, degradation, or matrix deposition) to the overall measured stiffness.

Cytochalasin D treatment (an actin generated active force inhibitor) of mature tumoroids was used to test what portion of overall stiffness was directly related to cell‐generated contractile forces (Figure 3F,G). The stiffness of the HT‐29 tumoroids significantly decreased (−25% compared to untreated control, *p* = 0.0003) after Cytochalasin D treatment. However, no significant difference was observed for the HCT 116 after treatment (*p* = 0.1374). This suggests that cell‐generated contraction has a significant contribution to the overall stiffness of HT‐29 (the low invasive cells) tumoroids only.

To investigate the contribution of ECM remodeling, the stiffness of tumoroids after decellularization was measured (Figure 3F,G). Stiffness of both HT‐29 and HCT 116 tumoroids significantly decreased after decellularization (by −50% for HT‐29, *p* < 0.0001 and by −37% HCT 116, *p* = 0.0001). Moreover, a significant decrease in stiffness was measured when comparing acellular gel stiffness and HT‐29 and HCT 116 decellularized gels (−29% for HT‐29, *p* = 0.0052 and by −38% HCT 116, *p* < 0.0001, Figure 3H). The significant softening of decellularized tumoroids for both cell types compared to intact acellular gels suggests critical involvement of enzymatic degradation during cell‐ECM interactions for both cell types, as indicated by MMP protein activity in both cell lines (Section 2.4).

Measurements of the proliferation rates by metabolic assay showed no significant changes in rates over time (Figure S2, Supporting Information). Single cell stiffness measurements indicated stiffness of the high invasive ‐HCT 116 cells to be significantly lower than HT‐29 cells (Figure S3, Supporting Information). This data suggest that neither proliferation rate nor single cell stiffness impact the observed stiffening of HT‐29 tumoroids or softening of HCT 116 tumoroids, consolidating the hypothesis that the observed stiffness changes are mainly due to active cellular force generation and matrix remodeling/degradation.

These results indicate that the changes in the stiffness of the cancer microenvironment originate from multi‐facetted simultaneous processes including protein deposition, protein cross‐linking, protein degradation, fiber alignment, compaction of matrix fibers, and pore size changes.^[^ ^58^, ^59^ ^]^ Furthermore, active contractile forces can also contribute to the stiffness of the tissue. Both HCT 116 and HT‐29 tumoroids go through enzymatic degradation, however in the HT‐29 tumoroid, the active cell‐generated forces out‐balances the degradation leading to an overall stiffer tumoroid after 21 days. Other parameters such as fiber alignment and pore size changes, mentioned above, can also contribute to the observed stiffness changes and would be interesting candidates to explore in the future.

### 2.4 MMP Broad Spectrum Inhibition Limits Matrix Remodeling and Cancer Cell Invasion

Several matrix degradation markers were tested to determine which ones correlate with the measured softening of the ECM (Figure 4A–D). Zymography on day 21 tumoroid media showed activity of matrix metalloproteinases^[^ ^22^ ^]^ MMP‐2 and MMP‐9 proteins in both cell types (Figure 4A). Interestingly, mRNA analysis revealed enhanced upregulation of both *MMP‐2* and *MMP‐9* (significant upregulation of *MMP*‐*9*, *p* = 0.0077) for HCT 116 tumoroids compared to HT‐29 (Figure 4B,C). Furthermore, expression level of *TIMP‐1* (an inhibitor of metalloproteinase‐1^[^ ^22^ ^]^) was significantly higher in the less invasive HT‐29 cells (*p* = 0.0199; Figure 4D).

To determine how MMP activity influences matrix remodeling, a broad‐spectrum inhibitor Batimastat (BB‐94) was used. This is because MMPs other than MMP‐2 and MMP‐9 also likely contribute to the matrix degradation process. Batimastat (BB‐94) targets MMP‐1, 2, 3, 7, 8, and 9 by directly binding to Zn^2+^ ions in the active site.^[^ ^26^ ^]^ BB‐94 concentration was optimized via a cell viability assay (Figure S4, Supporting Information). The efficiency of the drug was confirmed using zymography by checking for MMP activity (Figure S5, Supporting Information). When treating tumoroid samples with BB‐94, the stiffness of tumoroids from both cell types were not significantly changed compared to acellular controls (*p* > 0.9999, Figure 4E). We also confirm viability of cells within tumoroid samples after BB‐94 treatment over the course of 21 days using a live/dead Viability/Cytotoxicity stain (Figure S6, Supporting Information).

To investigate effects of MMP inhibition on cell invasion, the complex tumoroid model was employed (Figure 2C and Figure 4H) and the invasion of cancer cells from the artificial cancer mass (ACM) into the stroma was observed over 21 days for both the untreated and treated tumoroids. MMP inhibition significantly reduced area and distance of invasion for both the HT‐29 and HCT 116 cancer cells (Figure 4F,G). Quantification of the area of the invasion showed significant reduction after treatment (from 155 × 10^3^ µm^2^ to 9 × 10^3^ µm^2^ for HT‐29 cells, *p* = 0.0015 and from 522 × 10^3^ µm^2^ to 301 × 10^3^ µm^2^ for HCT 116, *p* = 0.0196, Figure 4F). Furthermore, for both cell types, the distance of invasion also decreased after treatment (from 130 to 21 µm for HT‐29 cells, *p* = 0.0042 and from 444 to 221 µm for HCT 116, *p* = 0.0079, Figure 4G).

We have shown that MMPs are strongly involved in the matrix degradation processes, and their effects are more pronounced in the highly invasive HCT 116 cancer cells. Interestingly, MMP inhibition also perturbed the stiffening behavior of less invasive HT‐29 cells (Figure 4E). It is reported that inhibiting MMPs may disturb multiple mechano‐bases mechanics in cells. In particular, broad spectrum MMP inhibition has been shown to limit cells ability to contract the matrix.^[^ ^60^ ^–^ ^63^ ^]^ This is consistent with our finding that active cell generated contractile forces are the major source of stiffening observed in HT‐29 tumoroids.

## 3 Conclusion 

Using a 3D cancer model of physiologically relevant stiffness, the role of cancer cells on changes in ECM mechanical properties was investigated by AFM indentation tests. We found that low invasive cancer cell lines stiffen their environment, and that the stiffening was partly attributed to cell generated contractile forces. The stiffening observed can be linked to early onset tumor characteristics where the tumor‐stroma interface stiffens, protecting the cancer cells from external factors such as immune cells and chemical signaling.^[^ ^5^ ^]^ Highly invasive cells, however, showed a significant softening of the ECM which likely facilitates cell migration and invasion in dense matrices by creating holes and paths for them. The degradation can mainly be attributed to the enzymatic activity of MMPs. Targeting MMP activity through broad‐spectrum drug inhibition stopped ECM softening. It also significantly reduced cancer cell invasion into the stroma. Our biomimetic 3D cancer model suggests a strong correlation between mechanical properties, invasion patterns and matrix degradation. Collectively, this point toward the potential effectiveness of MMP drugs as a mechano‐based cancer therapy.

## 4 Experimental Section

### Cell Culture

HT‐29 and HCT 116 immortalized colorectal cancer cell lines were obtained from the European Collection of Authorized Cell Cultures (Sigma–Aldrich, Dorset, UK). HDF were purchased from Invitrogen (Paisley, UK). All cells were cultured in Dulbecco's Modified Eagle Medium (DMEM) (low glucose DMEM for the cancer cells and high glucose DMEM for the HDF) supplemented with 10% FBS, 100 units mL^−1^ penicillin and 100 µg mL^−1^ streptomycin (all from Gibco^TM^ through Thermo Fisher Scientific, Loughborough, UK). All cell types were cultured at 5% carbon dioxide atmospheric pressure and at 37 °C and passaged regularly in 2D monolayers. HDFs were used at passage <10.

### Engineering of 3D Biomimetic Tumoroids

Tumoroids used in this study were termed “simple” and “complex.” Simple tumoroids (Figure 2B) consisted of 24‐well sized collagen gels with a cellular density of 2.7 × 10^6^ cells/sample, while complex tumoroids are compartmentalized consisting of a central ACM with a cellular density of 5 × 10^5^ cells/ACM surrounded by an acellular stroma (Figure 2C). Tumoroids were fabricated as previously described.^[^ ^40^ ^]^ Briefly, following the RAFT^TM^ protocol page 8–9, a collagen mixture was made as follows: 80% monomeric rat‐tail collagen type‐1 (First Link, Birmingham UK), 10% 10× MEM (Sigma–Aldrich, Dorset, UK), 6% neutralizing agent (N.A.), and 4% cells suspended in media. The N.A. was prepared from 17% 10 m NaOH (Sigma–Aldrich, Dorset, UK) and 1 m HEPES buffer (Gibco through Thermo Fisher Scientific, Loughborough). Collagen gels were cross‐linked at 37 °C for 15 min and plastic compression was conducted using the RAFT absorbers. Tumoroids were cultured to the desired timepoint at 5% CO_2_ atmospheric pressure and 37 °C in 1 mL of media per well. Fifty percent media changes were performed every 48 h to allow growth factors released by the cells to always be present.

### Ex Vivo Colorectal Tissue

Patient samples were obtained, with informed consent, from patients with colorectal tumors at the Royal Free Hospital, London, UK (ethics number 21/WA/0388). Fresh tissue was sectioned using a vibratome (7000smz‐2 Vibrotome, Campden Instruments, Loughborough, UK) with the following setting: frequency 60 Hz, amplitude 1.75 mm, thickness: 900 µm. AFM was performed on three different sections.

### Matrigel and Collagen Hydrogels

Corning Matrigel GFR Membrane Matrix (Thermo Fisher Scientific, Loughborough, UK) was set at 100% (protein concentration of 7.3 mg mL^−1^). Collagen 2 and 6 mg mL^−1^ were set using the RAFT protocol. Collagen 2 mg mL^−1^ was made using monomeric rat‐tail collagen type‐1 (First Link, Birmingham UK). Collagen 2 mg mL^−1^ was made using Corning rat tail Collagen I high concentration (8.38 mg mL^−1^) (Thermo Fisher Scientific, Loughborough, UK).

### Atomic Force Microscopy

Simple tumoroids to be used for AFM measurements were set in custom 3D‐printed PEEK (polyetheretherketone) rings the size of a 24‐well plate (PBH Engineering Ltd., Hertfordshire, UK), placed in a 35 mm Petri dish (Sigma–Aldrich, Dorset, UK). To measure stiffness, a CellHesion 200 AFM (JPK BioAFM, Bruker Nano GmbH, Berlin, Germany) was used. Measurements were performed at room temperature, in Leibovitz's L‐15 Medium, no phenol red (Gibco^TM^ through Thermo Fisher Scientific, Loughborough, UK). The AFM was first calibrated in liquid, on glass, to determine sensitivity and sum. The exact spring constant was measured prior to the experiment, when gluing the bead. The cantilever used was a RFESP‐75 (k ≈2 N m^−1^, Bruker, Berlin, Germany) with a 50 µm of diameter glued glass bead (Cospheric LLC, California, USA). Each sample was probed along a grid (4×4 map of 1500×1500 µm leading to a total of 16 measurements per sample). The set force was determined to ensure a 10–15 µm indentation, which is less than 10% of the thickness of our samples (150–200 µm).^[^ ^64^ ^]^ On compressed collagen, the set force was 700 nN. Using the JPK BioAFM SPM data processing software, the Hertz model was fitted to the collected force curves to determine the Young's Modulus E, assuming a Poisson ratio of 0.5.^[^ ^65^ ^]^

### Batimastat (BB‐94), Cytochalasin D, and Decellularization Treatments

For MMP inhibition, tumoroids were treated with 5 µm of Batimastat (BB‐94) every 48 h (Abcam, Cambridge, UK), diluted in media with 0.1% DMSO vehicle control. This concentration was optimized using a PrestoBlue Cell Viability assay (Invitrogen, through Thermo Fisher Scientific, Loughborough; Figure S4, Supporting Information). It was proven efficient using zymography to check for MMP activity (Figure S5, Supporting Information). Cytochalasin D (Invitrogen, through Thermo Fisher Scientific, Loughborough) was used at 20 µm diluted in media with 0.1% DMSO vehicle control and applied for 1 h. Decellularization was achieved using 0.5% triton X‐100 (Sigma–Aldrich, Dorset, UK) and 11 mm ammonium hydroxide (Sigma–Aldrich, Dorset, UK) in PBS for 1 h under gentle agitation.

### RNA Isolation and Quantitative Polymerase Chain Reaction (qPCR)

The primers used have previously been published or were designed according to the MIQE guidelines (**Table** **1**).^[^ ^66^ ^]^ RNA was extracted from complex tumoroids using the phase separation TRI Reagent and chloroform method.^[^ ^67^ ^]^ Complementary DNA was transcribed using the High‐Capacity cDNA Reverse Transcription Kit (Applied Biosystems through, Fisher Scientific, Loughborough, UK). The iTaq Universal SYBR Green Supermix (Applied Biosystems through, Fisher Scientific, Loughborough, UK) was used to amplify the gene of interest. Relative gene expression was calculated using the ∆Ct method, normalizing to glyceraldehyde 3‐phosphate dehydrogenase (GAPDH).^[^ ^68^ ^]^

**Table 1:** Primer sequences

	F’ sequence	R’ sequence
EpCAM (EJC Pape 2019^[^ ^40^ ^]^)	TTGCTGTTATTGTGGTTGTGGTG	CCCATCTCCTTTATCTCAGCCTTC
VIM	TCTCTGGCACGTCTTGACCTTG	CGATTTGGACATGCTGTTCCTG
MMP2 (SR Bakkalci 2021^[^ ^42^ ^]^)	CAGGAGGAGAAGGCTGTGTTC	TAAAGGCGGCATCCACTCG
MMP9	CAGTCCACCCTTGTGCTCTTCC	TTCGACTCTCCACGCATCTCTG
TIMP1 (BJC Pape 2020^[^ ^69^ ^]^)	TACTTCCACAGGTCCCACAACC	GCATTCCTCACAGCCAACAGTG
GAPDH (Al Hosni iScience 2022^[^ ^70^ ^]^)	GCTCTCTGCTCCTCCTGTTC	CGACCAAATCCGTTGACT CC

### Zymography

Media was collected from complex tumoroids after 21 days of cultivation and concentrated using Amicon Ultra‐2 Centrifugal Filter Units, of 30 kDa cutoff (Sigma–Aldrich, Dorset, UK). The media samples were mixed 1:1 with NovexTris‐Glycine SDS sample buffer, and left at room temperature for 5 min to active MMPs. A precast Novex 0.1% gelatine zymogram gel was loaded with X µL of sample and ran for 2 h at 125 V. The gel was then incubated in renaturing buffer for 30 min, developing buffer for 30 min and finally in fresh developing buffer overnight in a humidified incubator. The next morning, the gel was washed in DI water three times and stained for 2 h in SimplyBlue SafeStain. The gel was then imaged using an Epson Perfection V39 scanner (Epson, Nagano, Japan). All reagents were from Invitrogen, through Thermo Fisher Scientific, Loughborough, UK.

### Measurements of the Invasive Phenotype

Tumoroids were formalin fixed using 10% neutrally buffered formalin (Sigma–Aldrich, Dorset, UK) for 30 min and then washed and stored in phosphate buffered saline (PBS; Gibco, Thermo Fisher Scientific, Loughborough, UK). Images were taken using the Zeiss AxioObserver instrument and software (Zeiss, Oberkochen, Germany) on brightfield mode. Images were taken at four locations per samples as previously described.^[^ ^40^ ^]^ Distance and area of invasion were measured using Fiji ImageJ software.^[^ ^71^ ^]^

### Statistical Analysis

All data were analyzed and visualized using GraphPad Prism 9 software. The experiments were conducted with a minimum of *n* = 3. Usually, two or three technical replicates were also conducted and then averaged over. All data were first tested for normality using Shapiro–Wilk for *n* ≥ 3 or D'Agostino for *n* ≥ 7. Parametric tests used were unpaired Student's *t*‐test or a one‐way ANOVA. Nonparametric tests used were either Mann–Whitney or Kruskal–Wallis test. All *n* numbers, *p*‐values, and tests conducted are mentioned in figure caption. *P*‐value significance is indicated as: 0.05 < *, 0.005 < **, 0.0005 < ***, and 0.00005 < ****.

## Conflict of Interest

The authors declare no conflict of interest.

## Author Contributions

A.M. designed and completed all experimental work, analyzed all data, and wrote the manuscript. U.C. and E.M. designed and supervised the project, wrote the manuscript, and provided funds and equipment. J.P. was involved in experimental planning, data analysis, experimental optimization, and manuscript writing. D.B. helped with obtaining patient tissue and tissue sectioning. Y.J. and C.H. helped with AFM training and optimization. All authors approved the manuscript.

## Ethics Statement

All methods were carried out in accordance with relevant guidelines and regulations. Patient samples were obtained, with informed consent, from patients with colorectal tumors through the Tissue Access for Patient Benefit initiative at The Royal Free Hospital, London, UK. The ethics was approved by the University College London Royal Free Hospital BioBank Ethical Review Committee; Research Ethics Committee Reference number 21/WA/0388).

## Supporting information

Supporting Information

## Data Availability

The data that support the findings of this study are available from the corresponding author upon reasonable request.
