# Supplementary material for: Evaluating the Impact of a Biomimetic Mechanical Environment on Cancer Invasion and Matrix Remodeling
Source: Adv Healthc Mater. 2022 Nov 20;12(14):2201749. doi: 10.1002/adhm.202201749 (PMC11468596; doi:10.1002/adhm.202201749)
Supplement: Supplementary file 1 — Supporting Information [file ADHM-12-2201749-s001.pdf]

ADVANCED HEALTHCARE MATERIALS

Supporting Information

for *Adv. Healthcare Mater.*, DOI 10.1002/adhm.202201749

Evaluating the Impact of a Biomimetic Mechanical Environment on Cancer Invasion and Matrix Remodeling

Auxtine Micalet, Judith Pape, Deniz Bakkalci, Yousef Javanmardi, Chloe Hall, Umber Cheema and Emad Moeendarbary**

Supporting Information

Evaluating the Impact of a Biomimetic Mechanical Environment on Cancer Invasion and Matrix Remodelling

Auxtine Micalet[1,2], Judith Pape[2], Deniz Bakkalci[2], Yousef Javanmardi[1], Chloe Hall[1], Umber Cheema[2], Emad Moeendarbary[1*]*

[1] Department of Mechanical Engineering, University College London, Gower Street, WC1E 6BT, London, United Kingdom.

[2] Centre for 3D Models of Health and Disease, Department of Targeted Intervention, Division of Surgery and Interventional Science, University College London, Charles Bell House, 43-45 Foley Street, W1W 7TS, London, United Kingdom.

*These authors share senior authorship
Corresponding authors:
Umber Cheema: u.cheema@ucl.ac.uk
Emad Moeendarbary: e.moeendarbary@ucl.ac.uk

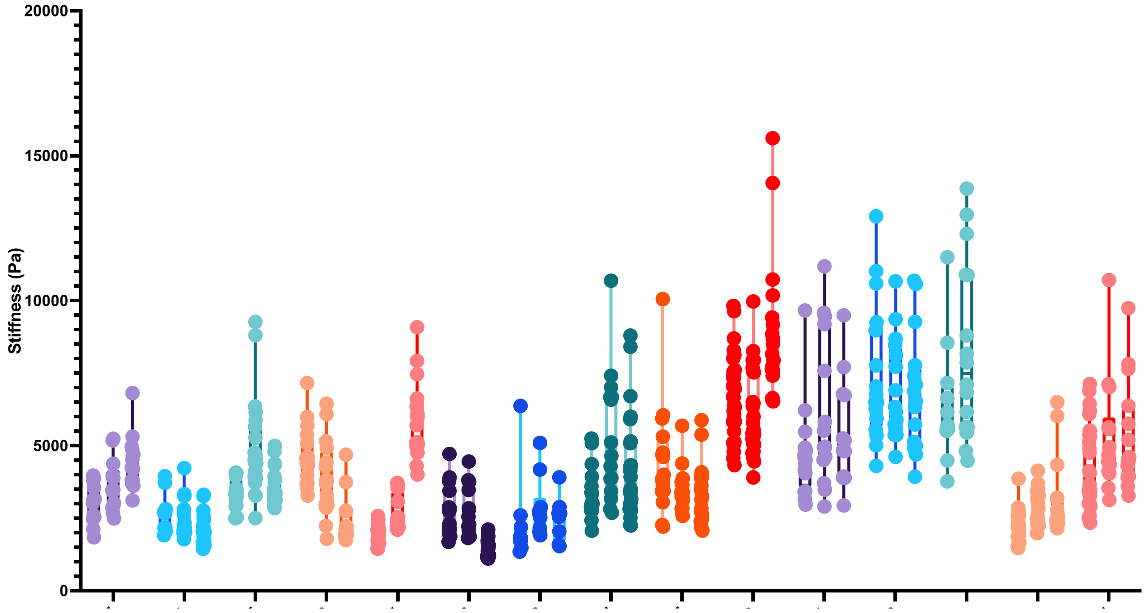

Figure S1- Stiffness (Pa) of all acellular controls made for this work. Each experiment was set with an n=3 of acellular controls, which are shown here as one colour. The stiffness varies within one sample but also from gel to gel and experiment to experiment due to the experimental process.

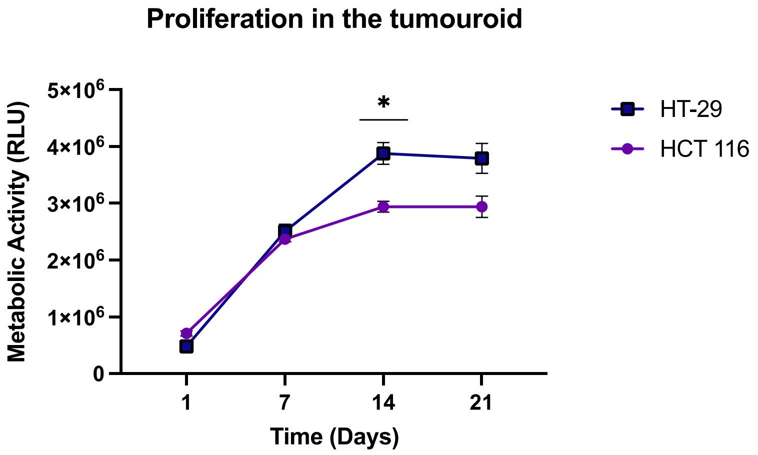

Figure S2- Metabolic activity of cancer cell lines HT-29 and HCT 116 in compressed collagen gel over 21 days using CellTiter Glo®. The proliferation is uncorrelated with stiffness of the tumouroids. If the 'soft' felt by AFM was due to a higher cell number, we would expect the HCT 116 to have a higher proliferation rate. All n=3, showing (mean ± SEM). Shows unpaired t test significance at each time point. All p-value significance is indicated as: 0.05<, 0.01<**, 0.001<*** and 0.0001<****.*

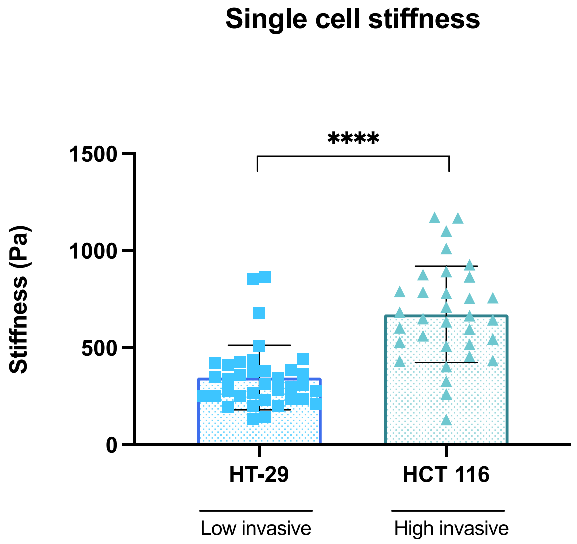

Figure S3- Stiffness (Pa) of HT-29 and HCT 116 single cells measured by AFM. Cells measured after 24 hours on a glass petri dish, with a cantilever of k ~0.1 N/m, a bead of 10 µm, and a force of 2 nN. All n>34, showing (mean ± SD) and showing Mann-Whitney significance (p<0.0001).

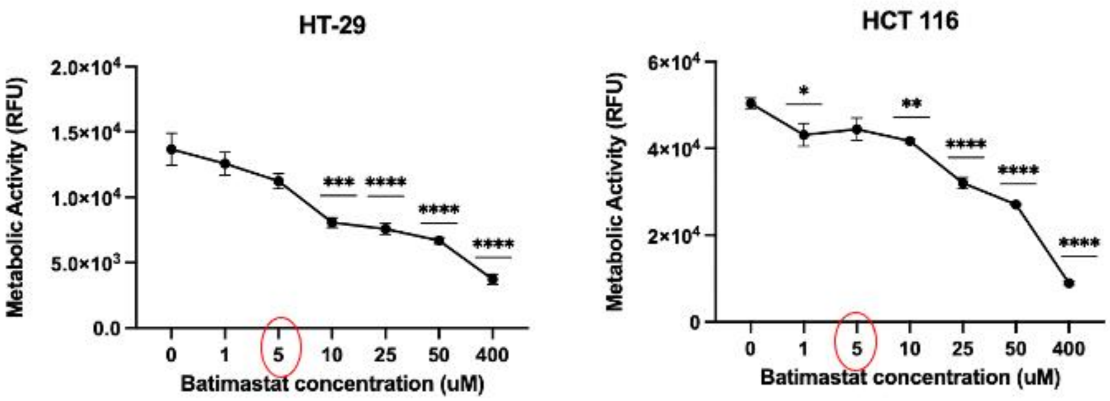

Figure S4- Cell viability assay in 2D for HT-29 and HCT 116 cells, treated with increasing concentrations of Batimastat in 0.1% DMSO (from 1 µM to 400 µM). 5 µM was chosen as the optimum concentration as it was it highest concentration possible without affecting cell viability.

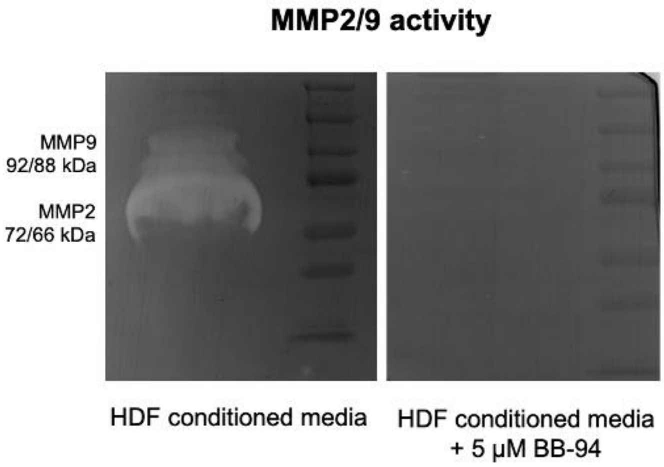

Figure S5- Zymography using human dermal fibroblast conditioned media. It shows MMP-2 and MMP-9 presence and activity in the untreated condition and no MMP activity when incubated with 5 µM of BB-94

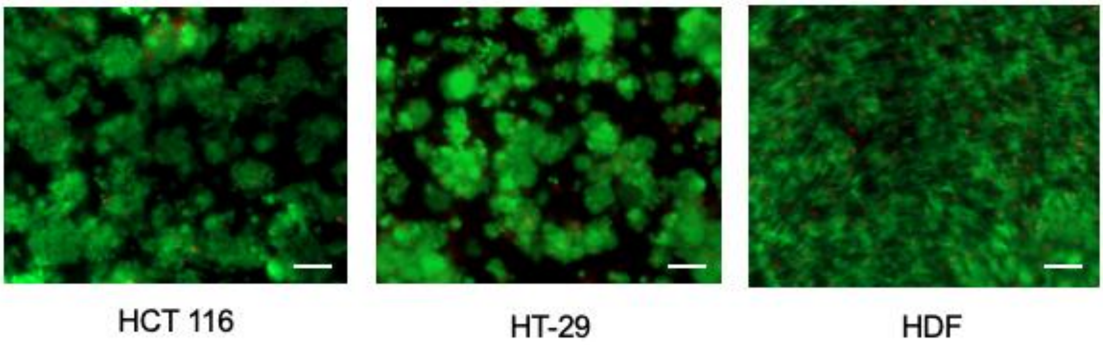

Figure S6- Live/dead cell viability staining of BB-94 treated tumouroids after 21 days. MMP inhibition via BB-94 treatment, at 5µM every 48 hours. Green = Live, Red = Dead. Scale bars = 100 µm
